# Resilience, predictor of empathy in Nursing students

**DOI:** 10.15649/cuidarte.4768

**Published:** 2025-12-17

**Authors:** Víctor P. Díaz-Narváez, Andrea Vallecampo Contreras, Johanna Campos de Chavarría, Nuvia Estrada-Méndez, Doris Alicia Sánchez de Elías, Lindsey W. Vilca, Alejandro Reyes-Reyes, José Gamarra-Moncayo

**Affiliations:** 1 Universidad Andres Bello. Santiago, Chile. Universidad Evangélica de El Salvador. E-mail: victor.diaz@unab.cl Universidad Andres Bello Santiago Chile victor.diaz@unab.cl; 2 Universidad Evangélica de El Salvador. San Salvador. El Salvador. E-mail: andrea.vallecampo@uees.edu.sv Universidad Evangélica de El Salvador Salvador andrea.vallecampo@uees.edu.sv; 3 Universidad Evangélica de El Salvador. San Salvador. El Salvador. E-mail: johanna.campos@uees.edu.sv Universidad Evangélica de El Salvador Salvador johanna.campos@uees.edu.sv; 4 Universidad Evangélica de El Salvador. San Salvador. El Salvador. E-mail: nuvia.estrada@uees.edu.sv Universidad Evangélica de El Salvador Salvador nuvia.estrada@uees.edu.sv; 5 Universidad Evangélica de El Salvador. San Salvador. El Salvador. E-mail: investigaciones.ciss@uees.edu.sv Universidad Evangélica de El Salvador Salvador investigaciones.ciss@uees.edu.sv; 6 Universidad Señor de Sipán. Chiclayo, Perú. E-mail: lwquiro@gmail.com Universidad Señor de Sipán Chiclayo Perú lwquiro@gmail.com; 7 Universidad Santo Tomás. Concepción, Chile. E-mail: areyesr@santotomas.cl Universidad Santo Tomás Concepción Chile areyesr@santotomas.cl; 8 Universidad Católica Santo Toribio de Mogrovejo. Chiclayo, Perú. Email: gamarramoncayoj@gmail.com Universidad Católica Santo Toribio de Mogrovejo Chiclayo Perú gamarramoncayoj@gmail.com

**Keywords:** Resilience, Psychological, Empathy, Psychometrics, Reproducibility of Results, Students, Vocational Education, Resiliencia Psicológica, Empatía, Psicometría, Reproductibilidad de Resultados, Estudiantes, Formación Vocacional, Resiliência Psicológica, Empatia, Psicometria, Reprodutibilidade dos Testes, Estudantes, Educação Vocacional

## Abstract

**Introduction:**

Studies attempting to predict empathy based on resilience are characterized by incomplete theories of both constructs and focus on obtaining empirical evidence.

**Objective:**

To verify whether resilience can predict empathy.

**Materials and Methods:**

A cross- sectional construct validity study was conducted. Salvadorean Nursing students were assessed using the Jefferson Scale of Empathy-Health Professions Students (JSE-HPS) and the Engineering, Ecological and Adaptive (EEA) resilience scale. Psychometric analyses (confirmatory factor analysis, reliability, and invariance) were conducted, and prediction was assessed using structural equations.

**Results:**

The compliance of the model of both constructs and the reliability of the data were verified. Some dimensions of resilience positively predicted the dimensions of empathy, while others predicted them negatively.

**Discussion:**

Ecological resilience and engineering resilience positively predicted all the dimensions of empathy. However, adaptive resilience negatively predicted empathy, suggesting that students may lack sufficiently developed adaptive traits to prevent declines in "compassionate care" and "standing in the patient's shoes." Therefore, their ability to connect emotionally and understand the patient's situation is hampered by a deficit of the traits that support adaptation to new situations.

**Conclusion:**

Empathy and resilience education cannot be independent of each other. On the contrary, resilience exerts a protective effect that enables the free expression of empathy that students have developed over the course of their lives.

## Resumen

**Introducción:**

Los estudios que intentan predecir la empatía a partir de la resiliencia se caracterizan por teorías incompletas de ambos constructos y por centrarse principalmente en la obtención de evidencia empírica.

**Objetivo:**

Comprobar si la resiliencia puede predecir la empatía.

**Materiales y métodos:**

Estudio de corte transversal y de validez de constructo. Se evaluó a estudiantes salvadoreños de enfermería mediante la Jefferson Scale of Empathy–Health Professions Students (JSE-HPS) y la Engineering, Ecological and Adaptive (EEA). Se llevaron a cabo análisis psicométricos (análisis factorial confirmatorio, confiabilidad e invarianza) y la predicción se evaluó mediante ecuaciones estructurales.

**Resultados:**

Se verificó el cumplimiento del modelo en ambos constructos y la confiabilidad de los datos. Algunas dimensiones de resiliencia predijeron positivamente las dimensiones de la empatía, mientras que otras lo hicieron de manera negativa.

**Discusión:**

La resiliencia ecológica y la de ingeniería predijeron positivamente todas las dimensiones de la empatía. Sin embargo, la resiliencia adaptativa predijo negativamente la empatía, lo que sugiere que los estudiantes no tienen los rasgos adaptativos suficientemente desarrollados para evitar disminuciones en “cuidado compasivo” y “ponerse en el lugar del paciente”. En consecuencia, su capacidad para conectarse emocionalmente y comprender la situación del paciente se ve limitada por un déficit en los rasgos que favorecen la adaptación a nuevas situaciones.

**Conclusiones:**

La formación en empatía y resiliencia no puede abordarse de manera independiente. Por el contrario, la resiliencia ejerce un efecto protector que permite la libre expresión de la empatía desarrollada por los estudiantes a lo largo de su vida.

## Resumo

**Introdução:**

Estudos que buscam predizer a empatia com base na resiliência são caracterizados por teorias incompletas de ambos os construtos e se concentram na obtenção de evidências empíricas.

**Objetivo:**

Testar se a resiliência pode predizer a empatia.

**Materiais e métodos:**

Foi realizado um estudo transversal de validade de construto. Estudantes de enfermagem colombianos foram avaliados por meio da Escala Jefferson de Empatia para Estudantes de Ciências da Saúde e da Escala de Resiliência Individual. Utilizou-se análise psicométrica (análise fatorial confirmatória, confiabilidade e invariância), e a predição foi realizada por meio de equações estruturais.

**Resultados:**

A conformidade do modelo de ambos os construtos e a confiabilidade dos dados foram verificadas. Constatou-se que algumas dimensões previram positivamente as dimensões da empatia, enquanto outras as previram negativamente.

**Discussão:**

A resiliência ecológica e a resiliência da engenharia, especificamente, predizem positivamente todas as dimensões da empatia. No entanto, a resiliência adaptativa a prediz negativamente. Esta última situação implica que os estudantes não possuem traços adaptativos suficientemente desenvolvidos para evitar um declínio nas dimensões do cuidado compassivo e de "colocar-se no lugar do paciente". Portanto, sua capacidade de se conectar emocionalmente e compreender a situação do paciente é prejudicada por um déficit nas características que permitem a adaptação à nova situação.

**Conclusões:**

O treinamento em empatia e resiliência não podem ser independentes. Pelo contrário, o efeito protetor da resiliência permite a livre expressão da empatia que o aluno desenvolveu ao longo da vida.

## Introduction

Empathy is an attribute that enables interaction between Nursing professionals and patients (intersubjectivity)^1^. Through this interrelationship, Nursing professionals are able to understand the subjectivity of patients' thoughts, comprehend intellectually or imaginatively their conditions, and experience patients' emotions as if they were their own, but without renouncing the principle of objectivity and avoiding emotional contagion^2^. The described situation gives patients the opportunity to feel that their health condition is understood, helping to establish deeper bonds with the Nursing professional^3^. This situation benefits patients, Nursing professionals, and the comprehensive therapeutic process. All these benefits have been extensively described in several studies^4^,^5^. Consequently, empathy is an important contributing factor, along with other factors, in establishing a solid foundation for the development and implementation of humane patient care^6^.

The emergence and development of empathy can only be explained from both an evolutionary perspective (phylogeny) and an individual's life experience (ontogeny)^7^. Empathy is an attribute characterized by genetic inheritance rooted in phylogenetic processes and by the extent to which this inheritance is expressed through ontogenetic processes^8^. While phylogeny provides the genetic possibility of developing empathy (genetic makeup), ontogeny determines if that possibility can reach various stages of development. This suggests that ontogenetic processes are directly related to the development of empathic capacity in an individual. The empathy ultimately "achieved" depends on numerous factors influencing development^9^, beginning in early childhood and continuing until the neural structures that enable the development of positive emotions (limbic system)^10^ and cognitive abilities (prefrontal and temporal cortex) are fully developed in young adulthood^11^. Both facets of empathy evolve in parallel. Therefore, empathy development is a process that begins naturally in early childhood and continues until the neural architecture of young adults is consolidated^12^. As a result, one of the last (and most important) windows of opportunity for cultivating empathy aligned with the professional role of nurses is during their university education.

There are two fundamental stages for ensuring that empathy education for Nursing students has a greater chance of success. The first stage involves conducting an empathy "diagnosis." This diagnosis involves evaluating empathy levels, including cognitive and emotional components, and identifying strengths and weaknesses during the diagnosis. It is followed by a parallel analysis that examines factors that could theoretically have a positive or negative impact on empathy, such as resilience. Such a diagnosis could lead us to recognize that an effective diagnosis of empathy not only depends on the empathy that students have developed throughout their lives before entering university, but also includes assessing how certain factors may contribute to explaining it. Consequently, a serious and responsible intervention would not only include all the necessary elements in the teaching-learning process to consolidate empathy education, but also the need to introduce the factors shown to be predictors of empathy into the aforementioned processes. In line with this rationale, the present study aimed to determine whether the dimensions of resilience can predict the dimensions of empathy in Nursing students.

## Materials and Methods

**Design**

This was a non-experimental, cross-sectional, psychometric study with construct validity.

**Population**

The study population comprised Nursing students enrolled in the Faculty of Health Sciences at the Universidad Evangélica de El Salvador (El Salvador) (n=160).

**Sample**

The sample consisted of 110 students assessed in May 2024, representing 68.75% of the total population. Although this sample was not randomly selected, it included almost the entire population; therefore, the results can be extrapolated to the population under study.

**Variables**

Resilience was considered the independent variable, and empathy the dependent variable.

**Eligibility criteria**

**Inclusion.** Students who voluntarily expressed their desire to participate in this research and signed the informed consent form were included.

**Exclusion.** Students who did not attend classes on the day of data collection or those who completed the instruments but did not sign the informed consent form were excluded from the study.

**Instruments**

**Individual resilience**

Trait resilience scale. The Engineering, Ecological, and Adaptive (EEA) resilience scale^13^ assesses three facets of resilience: engineering (items 1-4), ecological (items 5-8), and adaptive (items 9-12). This scale consists of 12 items rated on a 5-point Likert scale ranging from "Strongly disagree" (1) to "Strongly agree" (5). The EEA resilience scale has demonstrated satisfactory internal consistency and test-retest reliability (MacDonald's omega = 0.70–0.86; Cronbach's alpha = 0.68–0.82). Furthermore, this scale exhibits a stable cross-cultural factor structure, convergent and construct validity in relation to personality traits, and a positive contribution to clinical and non-clinical psychological health statuses^13^.

**Empathy**

Jefferson Scale of Empathy-Health Professions students (JSE-HSS)^14^,^15^. This scale comprises 20 items that measure empathy levels in health science students across various specialties. Items are rated on a 7-point Likert scale ranging from 1 (strongly disagree) to 7 (strongly agree). The scale measures three dimensions: compassionate care (CC; items 1, 7, 8, 11, 12, 14, 18, 19); perspective taking (PT; items 2, 4, 5, 9, 10, 13, 15, 16, 17, 20); and standing in the patient's shoes (SPS; items 3 and 6). PT and SPS dimensions constitute the cognitive component of empathy, whereas CC reflects the emotional component of this construct. The scale has demonstrated adequate internal consistency (α=0.78- 0.92) and appropriate correlations with other psychological variables^15^.

Both instruments underwent cultural adaptation through the following processes: translation and back-translation (translation from the original English to Spanish and from Spanish into English), expert panel review of the translation, and finally, pilot testing with 20 volunteer students from the study population to verify content comprehension.

**Procedure**

Students were assessed in classrooms, in a formal academic setting, using an online questionnaire. The instruments were administered by properly trained educators who ensured students' voluntary participation.

**Statistical analysis**

Descriptive statistics (mean, standard deviation, skewness, and kurtosis) were calculated for both variables and their dimensions. For continuous quantitative variables, normality was tested using the Kolmogorov-Smirnov test (K-S; n>50).

Confirmatory Factor Analysis (CFA) was performed using the robust maximum likelihood estimator in a multiple linear regression (MLR) analysis^16^,^17^, as the items had more than five response categories^18^. Model fit was evaluated using the following criteria: root mean square error of approximation (RMSEA < 0.08), standardized root mean squared error (SRMR < 0.08), comparative fit index (CFI > 0.95), and Tucker-Lewis Index (TLI > 0.95)^19^,^20^. Internal consistency of the scale was assessed using Cronbach's alpha^21^ and McDonald's omega coefficients^22^, with values > 0.70 considered acceptable^23^. All analyses described were conducted in R (RStudio interface) using the following packages: lavaan version 0.6- 17, psych version 2.4.1, semTools version 0.5-6, and MVN version 5.9. Statistical significance was set at p < 0.05 (α = 0.05). All data collected are freely available for access and consultation at OSFHOME^24^. 

**Ethical considerations**

This study was approved by the Research Directorate and the Health Research Ethics Committee of the Universidad Evangélica de El Salvador (CEIS-UEES), Minutes No. 018 of April 2024. Participating students considered minors (age < 18 years) completed the instrument only after prior parental consent was obtained. The study was classified as minimal risk.

## Results

The age data were normal (p >0.05). The sample represented 68.75% of the total population. The distribution by sex was 20.00% male (n= 22) and 80.00% female (n = 88). The mean age of male students was 23.59 years (SD = 3.92), whereas the mean age of female students was 22.89 years (SD = 4.72). Table 1 presents mean, standard deviation, skewness, and kurtosis estimates for each construct studied and its respective dimensions. The skewness and kurtosis estimates are within acceptable ranges.

**Table 1:** Descriptive results of the studied constructs and their corresponding dimensions. n=110

	Minimum	Maximum	M ± SD	Skewness	Standard error	Kurtosis	Standard error
Empathy	70	127	97.38 ± 13.851	0.200	0.230	-0.954	0.457
Compassionate care	8	53	31.66 ± 10.986	-0.591	0.230	-0.197	0.457
Perspective taking	27	70	59.25 ± 9.421	-1.184	0.230	1.440	0.457
Standing in the patient's shoes	2	14	6.46 ± 2.515	0.179	0.230	-0.113	0.457
Resilience	28	60	44.44 ± 8.208	0.158	0.230	-0.833	0.457
Engineering resilience	4	20	13.56 ± 3.974	-0.233	0.230	-0.758	0.457
Ecological resilience	4	20	16.15 ± 3.118	-1.037	0.230	1.588	0.457
Adaptative resilience	4	20	14.73 ± 3.332	-0.442	0.230	0.426	0.457

M= Mean; SD=Standard Deviation

**Measurement models**

The present study found that the scale of empathy presents adequate model fit indices (χ2 = 261.00; df= 165; p < 0.001; RMSEA=0.075, 90% CI [0.056 – 0.093]; CFI=0.90; TLI=0.88; SRMR = 0.099), supporting validity based on internal structure. Regarding reliability, all dimensions of the scale show acceptable internal consistency: perspective taking (ω = 0.90; α = 0.89), compassionate care (ω = 0.87; α = 0.84), and standing in the patient's shoes (ω = 0.52; α = 0.51). 

In relation to the resilience scale, this instrument also showed strong evidence of validity based on internal structure (χ2 = 79.55; df = 51; p = 0.006; RMSEA=0.076, 90% CI [0.037 – 0.110]; CFI=0.95; TLI=0.93; SRMR = 0.063). In addition, it showed adequate and acceptable reliability across all dimensions: Engineering (ω = 0.88; α = 0.87), ecological (ω = 0.84; α = 0.84), and adaptive (ω = 0.79; α = 0.79). All these results show that both measurement models (empathy and resilience) are adequately represented and are suitable for the structural model. 

**Explanatory model**

**Figure 1:**
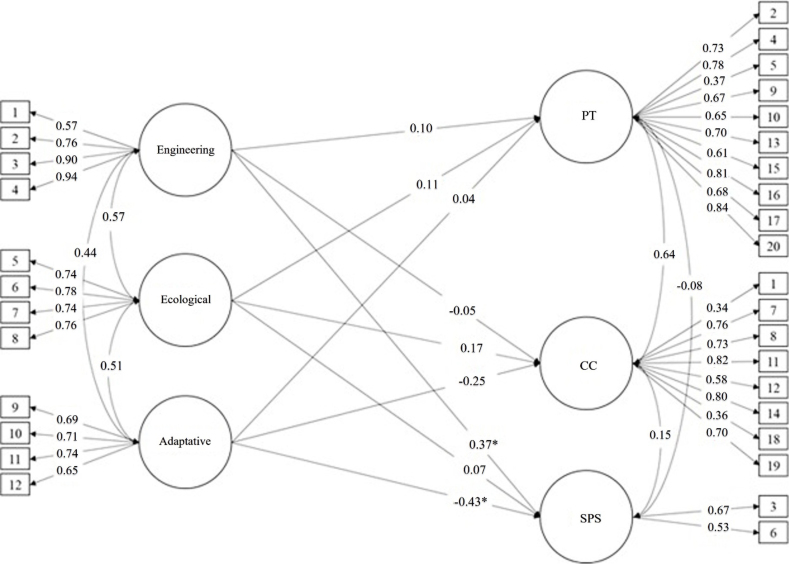
Explanatory model of empathy in Nursing students

The present study showed that the structural model presents acceptable fit indices (χ2 = 658.46; df= 447; p > 0.001; RMSEA=0.065, 90% CI [0.053 – 0.076]; CFI=0.88; TLI=0.86; SRMR = 0.086). As shown in Figure 1, the engineering resilience dimension did not significantly predict empathy dimensions, except for the "standing in the patient's shoes" dimension (0.37). Regarding the ecological dimension, Figure 1 shows that it did not significantly predict the dimensions of empathy. Furthermore, the adaptive dimension significantly predicted only standing in the patient's shoes dimension (0.43). 

*PT= Perspective Taking; CC= Compassionate Care; SPS= Standing in the Patient's Shoes. Values represent standardized regression coefficients (β) of Pearson's correlations.*

## Discussion

The results of the psychometric study confirmed that the data collected for both constructs demonstrated internal validity and reliability. Therefore, the analyses derived from these conditions will not be characterized by biases attributable to internal structure validity^25^. This process should be considered a standard methodological routine in all studies that employ measurement instruments to assess attributes, such as those analyzed in this study.

The concept of resilience generally expresses the personal and interpersonal capacities and internal strengths that enable learning and growth in the face of adverse circumstances. Some authors conceptualize resilience as a dynamic construct encompassing a broad range of phenomena that enable successful adaptation to threats that might otherwise hinder personal development^26^. Although resilience requires an individual response, it is not exclusively an individual characteristic since it is shaped by the interplay of individual and environmental factors. When these factors converge, they may themselves become sources of threat. In the context of Nursing education, students consistently encounter highly demanding situations that compel them to confront themselves. Such confrontation creates the conditions for students to recognize their potential and abilities, thereby strengthening themselves, learning, and responding effectively to disruptive circumstances encountered during professional practice^27^.

Within this general framework, the results reported in this article should be analyzed, and the analysis should include how resilience characteristics among Nursing students may predict empathy. In this regard, it should be emphasized that the three dimensions assessed by the resilience instrument represent three different but continuous moments. Partial success in one of them will not guarantee an adequate resilient response; rather, a positive resilient response depends on consistent success across all three moments. However, it should be noted that a positive resilient response does not necessarily translate into higher empathy, as the process of developing empathy follows its own path and distinct characteristics. The function of resilience is to cope with disruptions in such a way that the empathy attained by the student, whether high or low, is not affected.

Ecological resilience reflects the ability to resist and absorb a disturbance before reorganizing essential defense mechanisms to maintain equilibrium at critical levels. It is, therefore, the first response to a disturbance. The results of this study showed that ecological resilience positively predicted all three dimensions of empathy, suggesting that students may possess traits associated with the ability to endure negative events. Such traits include robustness, confidence in one's strengths and abilities, stoicism, resourcefulness, and determination in coping with negative events throughout life^28^,^29^.

These traits must be sufficiently strong so as not to affect any of the dimensions of empathy. This means that these traits associated with this dimension appear to have the property of not affecting the ability to engage emotionally and act to help the patient (CC); the ability to understand the patient's condition intellectually or imaginatively (PT); and the ability to appreciate the subjectivity of the patient's thoughts (SPS). It should be noted that the mean score observed for this dimension of resilience was 16.15 (Table 1), which indicates that there is room for considering the need to strengthen this dimension.

Engineering resilience refers to an individual's ability to recover or "bounce back" to baseline following adverse experiences^30^,^31^. Consequently, it is the ability to return to the initial state after suffering a negative event. It thus represents the "second moment" of resilient response. This dimension has been positively associated with "spirituality" and "emotional intelligence," some of the specific traits of this dimension^31^. In the present study, engineering resilience positively predicted AT and SPS (i.e., the cognitive component) but negatively predicted CC. However, the negative predictive value was low (Figure 1), suggesting that its effect is small. The observed mean score for this dimension was 13.56, which shows that there are still opportunities for further development in this dimension.

Adaptive resilience reflects the ability to adapt effectively to changes caused by disruption, adjust to circumstances, be flexible, change according to events, solve problems innovatively, constantly attempt to positively transform adverse aspects, and respond to disruptions with strength and moderation^32^,^33^. Adaptive resilience represents the "third moment" of resilient response. The results observed in relation to this dimension showed that adaptive resilience primarily predicted CC and SPS negatively. The relatively low mean score in this dimension (14.73; Table 1) may reduce students' emotional engagement at a given moment and, therefore, reduce their ability to assist patients. Moreover, it may diminish their ability to understand the subjectivity of patients' thoughts, thereby limiting their ability to feel and understand the patient's condition, hindering natural patient-student interactions, and, in the future, affecting the professional's natural activity with the patient.

Overall, the results observed in this study point to deficits in resilience education, particularly in adaptive resilience. These results are relevant to the professional future of Nursing students because they limit their chances of successfully coping with disturbances encountered during their Nursing practice^34^-^36^. In parallel, the empathy scores observed, when compared with established cut-off points for Latin American students^37^, suggest potential for further growth. Specifically, overall empathy scores and their dimensions were as follows: Empathy = 97.38 (high); CC = 31.66 (medium); AT = 59.25 (high), and SPS = 6.46 (medium). These results may hinder the educational initiatives aimed at cultivating the capacities required for humane patient care^37^-^39^.

Although not the primary objective of this study, it is necessary to point out that resilience, like empathy, can be enhanced through educational actions^40^-^43^. Therefore, these results should be considered by the institution responsible for the education of the participating students to improve their curriculum. Regardless of the specific findings of this study, the predictive role of resilience for empathy appears to be a general phenomenon^44^. Despite the scarcity of research of this type in Latin America, fostering education about resilience and empathy in Nursing students should be regarded as part of the social responsibility of higher education institutions^45^-^47^. Additionally, coping strategies for adverse events should also be taught.

**Strengths and limitations**

This study is characterized by an evaluation of the measurement model as a prerequisite for ensuring that the values of empathy and resilience (and their respective dimensions) are not biased by errors arising from non-compliance with the measurement model. As a result, the predictive values for some dimensions are robust compared to others. However, the sample cannot be considered representative of the target population, as the study characteristics did not allow for mandatory participation of students in completing the administered instruments.

## Conclusions

The findings indicate that the dimensions of resilience predict the dimensions of empathy. Nursing education institutions should therefore integrate resilience and empathy education into their curricula.
